# Draft Genome Sequence of a Granaticin-Producing Strain of Streptomyces parvus Isolated from a Roman Tomb in the Necropolis of Carmona, Spain

**DOI:** 10.1128/MRA.01127-19

**Published:** 2019-10-24

**Authors:** Jose L. Gonzalez-Pimentel, Valme Jurado, Leonila Laiz, Cesareo Saiz-Jimenez

**Affiliations:** a HERCULES Laboratory, Évora University, Évora, Portugal; b Instituto de Recursos Naturales y Agrobiologia de Sevilla (IRNAS-CSIC), Sevilla, Spain; Broad Institute

## Abstract

Streptomyces parvus strain C05 was isolated from the walls of a Roman tomb located in Carmona, Seville, Spain. Subsequent studies determined the capability of this strain for producing granaticins. Here, we report the 8.7-Mbp draft genome sequence for this bacterium.

## ANNOUNCEMENT

The phylum *Actinobacteria* is common in caves (1). Microclimatic conditions as well as the availability and nature of nutrients are critical elements for the biogeochemical processes carried out by different members of this group in subterranean environments (2–4), being especially relevant in the biodeterioration of cultural heritage (5).

The Circular Mausoleum is a small tomb located in the Roman Necropolis of Carmona, Spain. This tomb presents abundant and unusual violet stains affecting the mural paintings. A bacterium initially identified as *Streptomyces* sp. C05 was isolated from these stains and produced a violet pigment in the laboratory. Chemical analysis identified several granaticins as the source of the violet pigments (6). rRNA 16S and multilocus sequence analysis (MLSA) identified this bacterium as a strain of Streptomyces parvus (5). Granaticins exhibited antibacterial activity against Gram-positive bacteria and are synthesized by other *Streptomyces* species, such as S. vietnamensis and S. violaceoruber, but the type strain of *S. parvus*, NRRL B-1455, does not have the ability to synthesize granaticins (5, 7, 8).

Streptomyces parvus strain C05 was cultivated in tryptone soy agar incubated at 30°C. Genomic DNA isolation from this bacterium was carried out using the Marmur method (9) with modifications. The DNA concentration measurement was quantified with an Invitrogen Qubit 2.0 fluorometer. Genomic DNA was sequenced using 250-bp paired-end reads on an Illumina HiSeq platform by means of a Nextera XT library prep kit. A total of 2,183,823 reads were generated with a median insert size of 515 bp.

The read quality was checked with FastQC 0.11.5 (10). Reads were adapter trimmed using Trimmomatic 0.36 with a sliding window quality cutoff of Q15 (11). *De novo* assembly for this draft genome was performed using SPAdes 3.11.1 (12) with the “careful” option to reduce mismatches and short indels, and annotations were carried out using the NCBI Prokaryotic Genome Annotation Pipeline (13), with Sma3s (14) for functional annotations using the UniProt bacterium database with the “uniprot” option and antiSMASH (15) for secondary metabolite biosynthesis gene cluster prediction. A total of 729 contigs were obtained with a mean coverage of 29.94×. The *N*_50_ value was 28,163, and the largest contig was 102,255 bp. The genome size was 8,755,172 bp with an average G+C content of 71.4%. A total of 8,111 genes were detected, with 17 rRNA and 66 tRNA genes predicted.

*Streptomyces* sp. strain C05 was closely related to Streptomyces parvus NRRL B-1455^T^ (GenBank accession number VXCD00000000). The relatedness was assessed by calculating the average nucleotide identity with the BLAST and MUMmer algorithms using the JSpeciesWS Web service (16). Average nucleotide identity using BLAST (ANIb) and MUMmer (ANIm) between C05 and type strains presented values of 95.50% and 96.86%, respectively, suggesting that the *S. parvus* type strain and C05 belong to the same species.

The prediction of secondary metabolite clusters using antiSMASH corroborated the granaticin production since its genome presented 86% of the granaticin biosynthesis gene cluster described in *S. violaceoruber* (GenBank accession number AJ011500), in addition to 54 biosynthetic gene clusters involved in the production of different bioactive compounds. Therefore, the results obtained in former *in vitro* studies (5, 6), along with the prediction of secondary metabolites derived from in silico analysis carried out for Streptomyces parvus C05, promote the exploration of subterranean environments as a prolific source for biocompounds of biotechnological interest (17).

### Data availability.

This whole-genome shotgun project has been deposited at DDBJ/ENA/GenBank under the accession number VSZQ00000000. The version described in this paper is the first version, VSZQ01000000. BioProject and raw data are available at the accession numbers PRJNA562075 and SRR10059654, respectively.

## References

[1] Barton HA, Jurado V 2007 What’s up down there? Microbial diversity in caves. Microbe 2:132–138.

[2] Cuezva S, Fernandez-Cortes A, Porca E, Pašić P, Jurado V, Hernandez-Marine M, Serrano-Ortiz P, Hermosin B, Cañaveras JC, Sanchez-Moral S, Saiz-Jimenez C 2012 The biogeochemical role of *Actinobacteria* in Altamira Cave, Spain. FEMS Microbiol Ecol 81:281–290. doi:10.1111/j.1574-6941.2012.01391.x. 22500975

[3] Riquelme C, Hathaway JJM, Dapkevicius MLNE, Miller AZ, Kooser A, Northup DE, Jurado V, Fernandez O, Saiz-Jimenez C, Cheeptham N 2015 Actinobacterial diversity in volcanic caves and associated geomicrobiological interactions. Front Microbiol 6:1342. doi:10.3389/fmicb.2015.01342. 26696966 PMC4673402

[4] Diaz-Herraiz M, Jurado V, Cuezva S, Laiz L, Pallecchi P, Tiano P, Sanchez-Moral S, Saiz-Jimenez C 2013 The actinobacterial colonization of Etruscan paintings. Sci Rep 3:1440. doi:10.1038/srep01440. 23486535 PMC3595702

[5] Dominguez-Moñino I, Diaz-Herraiz M, Jurado V, Laiz L, Miller AZ, Santos JL, Alonso E, Saiz-Jimenez C 2017 Nature and origin of the violet stains on the walls of a Roman tomb. Sci Total Environ 598:889–899. doi:10.1016/j.scitotenv.2017.04.017. 28458206

[6] Diaz-Herraiz M, Laiz L, Jurado V, Miller AZ, Gonzalez-Perez JA, Santos JL, Alonso E, Saiz-Jimenez C 2016 Analytical pyrolysis evidences the presence of granaticins in the violet stains of a Roman tomb. J Anal Appl Pyrol 117:357–362. doi:10.1016/j.jaap.2015.10.018.

[7] Zhu HH, Guo J, Yao Q, Yang SZ, Deng MR, Phuong Le TB, Hanh VT, Ryan MJ 2007 *Streptomyces vietnamensis* sp. nov., a streptomycete with violet-blue diffusible pigment isolated from soil in Vietnam. Int J Syst Evol Microbiol 57:1770–1774. doi:10.1099/ijs.0.64774-0. 17684254

[8] Ichinose K, Bedford DJ, Tornus D, Bechthold A, Bibb MJ, Revill WP, Floss HG, Hopwood DA 1998 The granaticin biosynthetic gene cluster of *Streptomyces violaceoruber* Tü22: sequence analysis and expression in a heterologous host. Chem Biol 5:647–659. doi:10.1016/S1074-5521(98)90292-7. 9831526

[9] Marmur J 1961 A procedure for the isolation of deoxiribonucleic acid from microorganisms. J Mol Biol 3:208–218. doi:10.1016/S0022-2836(61)80047-8.

[10] Andrews S 2010 FastQC: a quality control tool for high throughput sequence data. https://www.bioinformatics.babraham.ac.uk/projects/fastqc.

[11] Bolger AM, Lohse M, Usadel B 2014 Trimmomatic: a flexible trimmer for Illumina sequence data. Bioinformatics 30:2114–2120. doi:10.1093/bioinformatics/btu170. 24695404 PMC4103590

[12] Bankevich A, Nurk S, Antipov D, Gurevich AA, Dvorkin M, Kulikov AS, Lesin VM, Nikolenko SI, Pham S, Prjibelski AD, Pyshkin AV, Sirotkin AV, Vyahhi N, Tesler G, Alekseyev MA, Pevzner PA 2012 SPAdes: a new genome assembly algorithm and its applications to single-cell sequencing. J Comput Biol 19:455–477. doi:10.1089/cmb.2012.0021. 22506599 PMC3342519

[13] Tatusova T, DiCuccio M, Badretdin A, Chetvernin V, Nawrocki EP, Zaslavsky L, Lomsadze A, Pruitt KD, Borodovsky M, Ostell J 2016 NCBI Prokaryotic Genome Annotation Pipeline. Nucleic Acids Res 44:6614–6624. doi:10.1093/nar/gkw569. 27342282 PMC5001611

[14] Muñoz-Mérida A, Viguera E, Claros MG, Trelles O, Pérez-Pulido AJ 2014 Sma3s: a three-step modular annotator for large sequence datasets. DNA Res 21:341–353. doi:10.1093/dnares/dsu001. 24501397 PMC4131829

[15] Blin K, Shaw S, Steinke K, Villebro R, Ziemert N, Lee SY, Medema MH, Weber T 2019 antiSMASH 5.0: updates to the secondary metabolite genome mining pipeline. Nucleic Acids Res 47:81–87. doi:10.1093/nar/gkz310. PMC6602434 31032519

[16] Richter M, Rosselló-Móra R, Oliver Glöckner F, Peplies J 2016 JSpeciesWS: a Web server for prokaryotic species circumscription based on pairwise genome comparison. Bioinformatics 32:929–931. doi:10.1093/bioinformatics/btv681. 26576653 PMC5939971

[17] Cheeptham N, Saiz-Jimenez C 2015 New sources of antibiotics: caves, p 213–227. *In* Sanchez S, Demain AL (ed), Antibiotics. Caister Academic Press, Norfolk, United Kingdom.

